# Opposing Effects of Intrinsic Conductance and Correlated Synaptic Input on *V_m_*-Fluctuations during Network Activity

**DOI:** 10.3389/fncom.2012.00040

**Published:** 2012-07-04

**Authors:** Jens Kolind, Jørn Hounsgaard, Rune W. Berg

**Affiliations:** ^1^Faculty of Health Sciences, Department of Neuroscience and Pharmacology, University of CopenhagenCopenhagen, Denmark

**Keywords:** fluctuations, balanced, inhibition, excitation, network, intrinsic properties, shunting

## Abstract

Neurons often receive massive concurrent bombardment of synaptic inhibition and excitation during functional network activity. This increases membrane conductance and causes fluctuations in membrane potential (*V_m_*) and spike timing. The conductance increase is commonly attributed to synaptic conductance, but also includes the intrinsic conductances recruited during network activity. These two sources of conductance have contrasting dynamic properties at sub-threshold membrane potentials. Synaptic transmitter gated conductance changes abruptly and briefly with each presynaptic action potential. If the spikes arrive at random times the changes in synaptic conductance are therefore stochastic and rapid during intense network activity. In comparison, sub-threshold intrinsic conductances vary smoothly in time. In the present study this discrepancy is investigated using two conductance-based models: a (1) compartment model and a (2) compartment with realistic slow intrinsic conductances. We examine the effects of varying the relative contributions of non-fluctuating intrinsic conductance with fluctuating concurrent inhibitory and excitatory synaptic conductance. For given levels of correlation in the synaptic input we find that the magnitude of the membrane fluctuations uniquely determines the relative contribution of synaptic and intrinsic conductance. We also quantify how *V_m_*-fluctuations vary with synaptic correlations for fixed ratios of synaptic and intrinsic conductance. Interestingly, the levels of *V_m_* -fluctuations and conductance observed experimentally during functional network activity leave little room for intrinsic conductance to contribute. Even without intrinsic conductances the variance in *V_m_* -fluctuations can only be explained by a high degree of correlated firing among presynaptic neurons.

## 1 Introduction

Changes in membrane potential in active neurons are caused by synaptic current generators activated by neurotransmitters and by voltage-activated intrinsic current generators. The relative contribution of synaptic and intrinsic current generators determines whether individual neurons during network activity, at one extreme, are weakly coupled autonomous oscillators (Toledo-Rodriguez et al., 2005; Grillner, 2006; Smith and Perrier, 2006) or in the other extreme driven by strong synaptic input. Since membrane currents are induced by conductance, their relative contribution to voltage changes scale with total membrane conductance. Synaptic input therefore has a divisive effect on the weight of intrinsic current generators to membrane potential. Therefore, when the synaptic input increases several-fold during network activity, the contribution of slow intrinsic conductance decreases by several-fold.

Synaptic integration is cell specific, influenced by morphology, input resistance, and active intrinsic response properties provided by voltage gated ion channels in the cell body and dendrites (Barret, 1975; Johnston and Wu, 1995; Koch, 1999; Williams and Stuart, 2003). For isolated neurons, the intrinsic response properties have a major role in controlling the activity patterns (Toledo-Rodriguez et al., 2005; Grillner, 2006; Smith and Perrier, 2006). For neurons in active networks however, the synaptic input can be so intense that the mean synaptic conductance is comparable to or larger than the input conductance of the neuron in absence of synaptic input. In this condition, the synaptic input itself severely distorts the electrotonic structure (Bernander et al., 1991; Korogod et al., 2000) and reduces the integration time up to ten-fold (Rapp et al., 1992; Koch et al., 1996; Berg et al., 2008). Furthermore, recent findings show that this level of synaptic intensity dampens or entirely eliminates the role of intrinsic properties in spike timing (Paré et al., 1998; Fellous, 2003; Kuhn et al., 2004; Berg et al., 2008;Fernandez and White, 2008, 2009; Berg and Hounsgaard, 2009). Whereas the intrinsic conductances may not always contribute detectably to spike patterns during network activity (Berg et al., 2008) they will certainly contribute to the total conductance and therefore affect the synaptically induced *V_m_*-fluctuations.

High-conductance states (Destexhe et al., 2003) occur not only in the neocortex (Destexhe and Paré, 1999), but also in the spinal cord during network activity (Alaburda et al., 2005; Berg et al., 2007). Excessive spiking and change in *V_m_* in these states are avoided by mixed inhibition and excitation (Gerstein and Mandelbrot, 1964; Shadlen and Newsome, 1994; van Vreeswijk and Sompolinsky, 1996; Gerstner and Kistler, 2005; Burkitt, 2006). Concurrent intensive and random inhibition and excitation causes the membrane potential (*V_m_*) to fluctuate in a stochastic fashion (Rudolph and Destexhe, 2005; Yarom and Hounsgaard, 2011). This state provides interesting computational properties and provides a unique cellular mechanism for gain control (Brunel et al., 2001; Chance et al., 2002; Destexhe et al., 2003; Fellous, 2003; Prescott and De Koninck, 2003; Burkitt, 2006). The amplitude of the fluctuations depends on the synaptic intensity. It is enhanced by correlated firing among presynaptic neurons (Stevens and Zador, 1998; Feng and Brown, 2000; Harsch and Robinson, 2000; Salinas et al., 2000; Svirskis and Rinzel, 2000; Stroeve and Gielen, 2001; Kuhn et al., 2003; Rudolph and Destexhe, 2006; Moreno-Bote et al., 2008) and curtailed by the input conductance (Rapp et al., 1992; Destexhe and Paré, 1999; Kuhn et al., 2004; Berg et al., 2008). The experimentally observed high-conductance state has primarily been attributed to synaptic activity, but slowly changing intrinsic conductances also contribute (Guillamon et al., 2006). Nevertheless, these two sources of sub-threshold conductance have distinct features. The dynamics of the macroscopic intrinsic conductance is slow and gradual compared with the abrupt conductance changes with synaptic transmission (Jacobson et al., 2005). The amplitude of the *V_m_*-fluctuations caused by voltage sensitive ion channels shifting between open and closed states (Diba et al., 2004; Jacobson et al., 2005) is an order of magnitude lower than *V_m_*-fluctuations caused by discrete inhibitory and excitatory events that involve opening and closing thousands of transmitter gated channels (Rudolph and Destexhe, 2003). For these reasons the relative contribution from non-fluctuating intrinsic conductance and fluctuating synaptic conductance in the high-conductance state can be determined if the cell is approximated as a single compartment. The goal of the present study is to estimate from the size of the synaptic fluctuations, what the relative roles of the intrinsic and synaptic conductances are compared to that of the mean synaptic conductance.

The intrinsic response properties of motoneurons are thought to play a crucial role in the translation of synaptic input to firing patterns in motor axons (Delgado-Lezama and Hounsgaard, 1999; Russo and Hounsgaard, 1999; Rekling et al., 2000; Grillner, 2006). However, recent experimental findings shows that spinal motoneurons, during functional network activity, can enter a high-conductance state in which firing is determined by synaptically induced fluctuations in membrane potential rather than intrinsic membrane properties (Alaburda et al., 2005;Berg et al., 2007, 2008). For this reason we base our analysis of the effect of intrinsic and synaptic conductance on fluctuations in membrane potential on experimental data from spinal motoneurons. However, our qualitative conclusions are valid for neurons in general.

We have previously determined how average conductance and *V_m_*-fluctuations varies in spinal motoneurons during functional network activity in the turtle (Berg et al., 2007, 2008; Jahn et al., 2011). With these experimental boundary conditions we investigate this regime and analyze how total conductance and the relative contribution of intrinsic and synaptic conductance affect *V_m_*-fluctuations. We also explore the effect of synaptic correlations on the *V_m_*-fluctuations at different levels of intrinsic conductance and compare with data from turtle motoneurons during functional network activity.

## 2 Materials and Methods

The analysis is primarily based on a 1-compartment (1C) generic model with conductance-based inhibitory and excitatory synaptic input arriving as two independent poisson processes with constant rates (Burkitt, 2006). This analysis is subsequently extended in a 2-compartment model which has previously been developed with more realistic intrinsic conductances for turtle motoneurons (Booth et al., 1997). For the 1C model, the membrane potential is held at a constant mean of −55 mV by balancing inhibition and excitation and the intrinsic conductance. The parameters of the model are based on experimental data from hip-flexor motoneurons in adult turtles (Berg et al., 2008). In the 1C model we assume that intrinsic conductances are voltage insensitive in the range covered by *V_m_*-fluctuations around the mean. The steady-state conductance near the resting membrane potential is constant in turtle motoneurons (Delgado-Lezama et al., 1997). In addition, none of the transient membrane conductances have sufficiently steep voltage sensitivity and fast kinetics to contribute significantly to sub-threshold *V_m_*-fluctuations. This is supported by the finding that the change in average conductance during network activity is independent of voltage (supplement in Berg et al., 2007).

### 2.1 Conductance in the model

In order to analyze the relative roles of synaptic and intrinsic conductance, we will consider four generic types of conductance: synaptic and non-synaptic depolarizing conductances [equation (1)] with reversal potentials far depolarized from the resting membrane potential and synaptic and non-synaptic hyperpolarizing conductances [equation (2)] with reversal potentials hyperpolarized from the resting membrane potential. Secondly, on the time scale considered here the synaptic conductances are rapidly fluctuating while the intrinsic conductances are constant. In this way intrinsic conductances are treated as simple additions to the leak conductance. This can be expressed explicitly as

(1) $$G_{D}=G_{Syn.D}+G_{Int,D}$$

(2) $$G_{H}=G_{Syn.H}+G_{Int,H}$$

where *G_D_* is the total depolarizing conductance consisting of a synaptic part, *G_Syn,D_*, and an intrinsic part *G_Int,D_*. Similarly, *G_H_* is the total hyperpolarizing conductance, composed of a synaptic part, *G_Syn,H_* and an intrinsic part *G_Int,H_*. For simplicity we assume the synaptic and intrinsic currents have same reversal potential, *E_H_* and *E_D_*, for the hyperpolarizing and depolarizing conductances, respectively. In all simulations the average membrane potential is held constant and it does not exceed the two reversal potentials. Under these conditions the qualitative behavior of the model is not affected by choosing the same reversal for synaptic and intrinsic currents. In this simple model we can keep the total conductance constant, and the mean membrane potential fixed while changing the relative contribution of synaptic versus intrinsic conductances. It is useful to define a parameter, γ, which takes values between 0 and 1, where γ = 0 represents 100% intrinsic conductance and γ = 1 represents 100% synaptic conductance:

(3) $$\gamma =\frac{G_{Syn}}{G_{Syn}+G_{Int}}$$

where

(4) $$G_{Syn}=G_{Syn,D}+G_{Syn,H}$$

and

(5) $$G_{Int}=G_{Int,D}+G_{Int,H}$$

We further assume the synaptic fraction also applies for the hyperpolarizing and depolarizing conductances individually, i.e., *G_syn,D_* = γ*G_D_* and *G_syn,H_* = γ*G_H_*, which is equivalent to enforcing $G_{Syn,D}∕G_{D}=G_{Syn,H}∕G_{H}.$

### 2.2 Membrane equation for 1C model

In a 1C model of a single neuron the membrane potential is described by the current flow across the membrane via Ohms and Kirchoffs laws:

(6) $$C\frac{dV_{m}}{dt}=G_{L}\left(E_{L}-V_{m}\right)+G_{D}\left(E_{D}-V_{m}\right)+G_{H}\left(E_{H}-V_{m}\right)$$

where the three conductances are the leak, *G_L_*, with reversal potential *E_L_*, *G_H_*, and *G_D_*, as defined above. In order to avoid spiking, we keep the membrane potential in a balanced state of excitation and inhibition and do not include spiking mechanism in the model. The membrane equation can then be rewritten approximately as (see Kuhn et al., 2004):

(7) $$\left\langle \tau_{eff}\right\rangle \frac{dV_{m}}{dt}\approx \left\langle V_{m}\right\rangle -V_{m}$$

where

(8) $$\langle \tau_{eff}\rangle =\frac{C}{G_{tot}}$$

(9) $$G_{tot}=G_{L}+\langle G_{D}\rangle +\langle G_{H}\rangle$$

(10) $$\langle V_{m}\rangle =\frac{G_{L}E_{L}+\langle G_{D}\rangle E_{D}+\langle G_{H}\rangle E_{H}}{G_{tot}}$$

and 〈…〉 denotes time-averaged values. 〈*V_m_*〉 denotes the steady-state mean membrane potential in the balanced state. Note that equations (7) and (8) are only approximately valid since *C*/〈*G_tot_*〉 is the first order approximation of 〈τ*_eff_*〉 (Kuhn et al., 2004). If *G_L_* is kept constant then there is a direct relation between *G_H_* and *G_D_*, which comes from the balanced condition [equation (10)]. This relation is explicitly written as

(11) $$\left\langle G_{H}\right\rangle =\frac{G_{L}\left(E_{L}-\left\langle V_{m}\right\rangle \right)+\left\langle G_{D}\right\rangle \left(E_{D}-\left\langle V_{m}\right\rangle \right)}{\left\langle V_{m}\right\rangle -E_{H}}$$

Hence, the total conductance in the model is varied by changing 〈*G_D_*〉 and calculating what the 〈*G_H_*〉 should be in the balanced condition (*V_m_* = −55 mV). These values of are independent on the choice of γ, since γ determines the fraction of synaptic input and therefore the rate of input (see Section 5). Then lastly, 〈*G_tot_*〉 is calculated according to equation (9).

### 2.3 Parameters of 1C model

For comparison with the fluctuations recorded experimentally, parameters from the adult turtle spinal cord were used in all simulations. A capacitance of 806 pF and leak conductance of 64 nS was used for the passive membrane. This was the average measured in 32 motoneurons (see Results). For the reversal potentials *E_L_* = −75 mV, *E_H_* = −80 mV, and *E_D_* = 0 mV were used. The parameters for synaptic input were chosen based on voltage-clamp data. The median time constant and maximum conductance of multiple events was selected for the simulations. For excitation the α-synapse was specified by τ*_E_* = 2.4 ms and *g_max,E_* = 0.43 nS. For inhibition the α-synapse was specified by τ*_I_* = 5.5 ms and *g_max,I_* = 1.3 nS. The experimentally verified parameters for turtle motoneurons (see Results) are significantly lower than what was used in the study by Kuhn et al. (2004).

### 2.4 Balanced vs. concurrent inhibition and excitation

In the present paper we reserve the term *balanced inhibition and excitation* for the situation in which changes in synaptic intensity is performed so that 〈*V_m_*〉 is kept constant, i.e., by adjusting the ratio of inhibition and excitation appropriately. We use the term *concurrent inhibition and excitation* for the state in which the ratio between inhibition and excitation is kept constant while varying the synaptic intensity. Nonetheless, these two situations are concerning synaptic input, but in most of our analyses, intrinsic conductances are also present. This inclusion of intrinsic conductance compels redefinition of *balanced* and *concurrent* input to include the intrinsic depolarizing and hyperpolarizing conductances. Thus, if we define the ratio β of depolarizing and hyperpolarizing conductance β = *G_D_*/*G_H_* we can rewrite the equation (10) in terms of β as:

(12) $$\left\langle V_{m}\right\rangle =\frac{\left(\beta E_{D}+E_{H}\right)\left\langle G_{H}\right\rangle +G_{L}E_{L}}{G_{tot}}$$

The *balanced* and *concurrent* conditions are approximately equivalent for certain circumstances, as illustrated in the following. If *G_tot_* = *G_L_* ⇒ 〈*V_m_*〉 = *E_L_*. As *G_tot_* becomes larger (*G_tot_* → ∞), the leak conductance is diluted and 〈*V_m_*〉 approaches a constant value: 〈*V_m_*〉 = β*E_D_* + *E_H_*/β + 1. In the situations where the membrane potential is kept constant (〈*V_m_*〉 = −55 mV), β will approach an asymptotic value of β = 0.45 as the conductance increases. Here, the hyperpolarizing and depolarizing input are both *balanced* and *concurrent*. However, in most cases an increase in intensity at constant β will result in an increase of the membrane potential if β > (80/75) - 1, i.e., β > 0.067 or if the resting potential is below *E_L_* during a negative current injection. Qualitatively this means that under most circumstances (where β > 0.067) any increase in intensity with *G_D_*/*G_H_* constant will also result in an increase in membrane potential (see below and Figure 2).

### 2.5 Expected fluctuations in 1C model

Now we consider the second moment of the membrane potential, i.e., the variance and the standard deviation (σ). The opening of a single synapse is modeled as a conductance change following an α-function:

(13) $$g_{syn}\left(t\right)=\frac{t}{\tau }g_{max}\exp\left(1-\frac{t}{\tau }\right)$$

where *g_max_* is the maximal conductance from a single post-synaptic input, τ is the characteristic time constant of the synaptic input. These constants are determined experimentally. Inserting this into the general formulation of the membrane equation gives (Kuhn et al., 2004):

(14) $$\left\langle \tau_{eff}\right\rangle \frac{dV_{m}}{dt}=\left\langle V_{m}\right\rangle -V_{m}+\frac{tg_{max}e^{1-\frac{t}{\tau }}\left(E_{syn}-V_{m}\right)}{\tau G_{tot}}$$

where *E_syn_* is the reversal potential for the synaptic input, which is either excitatory or inhibitory (*E_D_* or *E_H_*). The membrane potential change from opening a single α-synapse can be approximated by the following expression if the change in voltage is small compared with the reversal potentials:

$$\begin{array}{rcll} V_{m,PSP}\left(t\right) & \approx \left\langle V_{m}\right\rangle +\left[E_{syn}-\left\langle V_{m}\right\rangle \right]\frac{g_{max}e}{C\tau } & & \\ & \;\left[\frac{-te^{-\frac{t}{\tau }}}{\frac{1}{\tau }-\frac{1}{\left\langle \tau_{eff}\right\rangle }}+\frac{e^{-\frac{t}{\left\langle \tau_{eff}\right\rangle }}-e^{-\frac{t}{\tau }}}{{\left(\frac{1}{\tau }-\frac{1}{\tau_{eff}}\right)}^{2}}\right] & & \text{(15)} \end{array}$$

Note in equation (15) that when τ*_eff_* decreases because of an increase in the total conductance, the amplitude of the PSP also decreases. Excitatory and inhibitory synaptic input is generated by two independent Poisson processes with rates λ*_E_* and λ*_I_*, which is also referred to as a shot-noise stochastic process (Rudolph and Destexhe, 2006). In this situation the variance of the membrane potential can be estimated from the single excitatory and inhibitory post-synaptic potential waveforms (EPSP and IPSP) using Campbells Theorem (Mathieson, 1977; Kuhn et al., 2004; Rudolph and Destexhe, 2006):

(16) $$\sigma^{2}=\lambda_{E}\int_{0}^{\infty }\;{\left(EPS\;P-\left\langle V_{m}\right\rangle \right)}^{2}dt+\lambda_{I}\int_{0}^{\infty }\;{\left(IPS\;P-\left\langle V_{m}\right\rangle \right)}^{2}dt$$

where 〈*V_m_*〉 is subtracted to get the integration over full the shape of PSPs. This relationship [equation (16)] predicts the behavior of the computational model. First, the variance initially increases with synaptic intensity since the input rates (λ*_e_* and λ*_i_*) increase. Secondly, since the evoked PSPs decrease with larger conductance [equation (15)] (see Kuhn et al., 2004; Moreno-Bote and Parga, 2005) the integrals in [equation (16)] will get smaller with increasing conductance. Therefore we expect the variance to first increase and then decrease at some point as a function of conductance. Now, in order to take the intrinsic conductance into account we first look at the mean synaptic conductance. The mean conductance from α-synaptic inputs arriving with rate λ*_j_* is,

(17) $$\left\langle G_{syn,j}\right\rangle =\lambda_{j}\tau_{j}eg_{max,j}$$

where *e* is the exponential number (Kuhn et al., 2004) and the index *j* represents either excitation (depolarizing) or inhibition (hyperpolarizing). Since γ indicates the fraction of synaptic conductance (*G_syn,D_* = γ*G_D_* and *G_syn,H_* = γ*G_H_*), the synaptic input rates can be expressed in terms of γ and the mean conductances:

(18) $$\lambda_{E}=\frac{\gamma \langle G_{D}\rangle }{\tau_{E}eg_{\max,E}}$$

(19) $$\lambda_{I}=\frac{\gamma \langle G_{H}\rangle }{\tau_{I}eg_{\max,I}}$$

Since the fraction γ only affects the synaptic input rates and not the post-synaptic-potential (PSP) waveform in Campbells theorem, we therefore see that the variance of the membrane potential is proportional to γ:

(20) $$\sigma^{2}\propto \gamma$$

The largest fluctuations in the membrane fluctuations occur when γ = 1, which is where the overall conductance consists of 100% synaptic conductance and 0% intrinsic conductance. The special case where *G_syn_* > 0 is kept constant the fluctuations would decrease asymptotically in size with zero as the limit when increasing *G_tot_*. If *G_syn_* = 0 there would be no fluctuations for any value of *G_tot_*.

Since there is a linear relationship between the input rates, λ*_E_* and λ*_I_*, σ^2^ and the membrane conductance [equations (16), (18), and (19)], with γ as one of the multiplicative factors, we expect the same but scaled-down shape of curve in a graph between *G_tot_* and the fluctuation size for decreasing values of γ.

### 2.6 Synaptic coincidence, κ, in 1C model

The prevailing irregularity of presynaptic spiking and the correlation among synaptic input are critical factors for the amplitude of fluctuations in *V_m_* (Stevens and Zador, 1998; Harsch and Robinson, 2000; Salinas et al., 2000; Svirskis and Rinzel, 2000; Stroeve and Gielen, 2001; Moreno et al., 2002; Rudolph and Destexhe, 2006; Moreno-Bote et al., 2008; El Boustani et al., 2009). For convenience, we introduce correlations in the timing of synaptic input in our model of uncorrelated Poisson-type synaptic input by letting multiple synaptic inputs arrive in perfect synchrony. In general terms, the degree of overall correlation is given by the parameter κ, which is a metric of how many synapses are active at the same time. The Greek letter κ is chosen to implicate that it is a parameter for *coincidence* input. For instance at κ = 2, a single synapse will never be active alone, but always in concert with another synapse and the input rate is half of what it would be at κ = 1. With α-synapses, a correlation of κ is equivalent to stronger synapses with *g_max′_* = κ*g_max_* arriving at a slower rate of λ′ = λ/κ. The unitary conductance *g_max_* is the conductance for the synaptic connection from one synaptic event in one neuron. Replacing (λ, *g_max_*) with (λ′, *g_max′_*) in Campbells theorem for the variance of the membrane potential [equations (16–19)] we see that fluctuations are proportional to the coincidence factor κ

(21) $$\sigma^{2}\propto \kappa$$

which has previously been described using a multichannel shot-noise approach (Rudolph and Destexhe, 2006). We therefore expect the standard deviation to be proportional with the square root of the coincidence factor. The full analytical expression for σ^2^ as a function of κ can be derived by inserting above in equation (16). Futhermore, we expect the graph of σ versus *G_tot_* to have the same but scaled-up shape when increasing κ, because of equation (21).

### 2.7 Coincidence factor vs. correlation

Using the coincidence factor (κ) has the advantage that no assumptions about the number of presynaptic neurons and the quantal release are necessary. However, to compare with other studies (see, e.g., Destexhe and Paré, 1999; Salinas et al., 2000; Rudolph and Destexhe, 2006; Moreno-Bote et al., 2008) our coincidence factor should be related to the correlation measure, referred to as ρ. ρ is the probability of Neuron A firing at time point *t_i_* given that Neuron B fires at time point *t_i_*. We can express ρ as the number of pair-wise correlated inputs (*C*) divided by the maximum number of pair-wise correlated inputs (*C_max_*). In a presynaptic network of *N* cells, firing is fully correlated if all *N* neurons fire simultaneously. In this situation Neuron A fires simultaneously with the *N* − 1 remaining neurons. Neuron B also fires simultaneously with *N* − 1 neurons, but to avoid double counting the maximum number of pair-wise correlated input is expressed as:

(22) $$C_{max}=\overset{N}{\underset{n=1}{\sum }}\;n-1=\frac{\left(N-1\right)N}{2}$$

In our model κ expresses how many neurons are firing at once. At κ = 2, the *N* neurons are firing as *N*/2 pairs. At κ = 3, the N neurons are firing as *N*/3 assemblies of 3 neurons. The number of pair-wise correlated inputs can be expressed from κ:

(23) $$C=\frac{N}{\kappa }\overset{\kappa }{\underset{i=1}{\sum }}\;i-1=\frac{\left(\kappa -1\right)N}{2}$$

We can now approximate the degree of correlation (ρ) from the coincidence factor (κ) and the number of presynaptic neurons (N):

(24) $$\rho =\frac{C}{C_{max}}=\frac{\kappa -1}{N-1}$$

The input rate to the motoneuron is given by the number of presynaptic neurons times their average firing rate, λ = *N*〈λ*_presynaptic_*〉. We assume that the presynaptic population fires at an average rate of 〈λ*_presynaptic_*〉 = 10 Hz. So, if each motoneuron receives 1 kHz input we assume *N* = 100 neurons. In all simulations we introduce the same level of correlation within the excitatory and inhibitory presynaptic populations. Inhibition and excitation is considered to be uncorrelated.

### 2.8 Booth–rinzel–kiehn 2C model

In order to verify our findings from the 1C model in a more biophysically realistic model, we used the established Booth–Rinzel–Kiehn (BRK) model (Booth et al., 1997). The BRK model is a 2-compartment (2C) model with intrinsic conductances representing the dynamics of turtle motoneurons (Figure 7A), and therefore appropriate for our investigation. The original published model parameters were also used in our study. The neuron was amended with time-varying inhibitory and excitatory conductances in the soma and dendritic compartment. The conductance time series were generated by adding Poisson-distributed α-synapses in the same way as described for the 1C model (see above). Reversal potentials and time constants were set at the same levels as for the 1C model. The maximum synaptic conductances were adjusted to give IPSPs and EPSPs at rest with same magnitude as in the 1C model. In the comparison to the 1C model, synaptic input was distributed according to the size of the compartments in the BRK model (90% on dendrite, 10% on soma), to get the same input rate per membrane area. The added intrinsic conductance was applied by proportionally increasing the intrinsic conductances of the BRK model (*G_L_*, *G_Ca–*N*_*, *G_Ca–*L*_*, *G_K(*Ca*)_*, *G_K–*dr*_*, *G_Na_*). To test for the impact of input distribution between the compartments, a fixed input rate was chosen (λ*_E_* = 3.2 Hz, λ*_I_* = 1.9 Hz; same as the rates at peak on Figure 7B) and then distributed in various proportions between soma and dendrite. To prevent spiking, a hyperpolarizing current (*I_app_*) was added in the soma compartment to keep the average membrane potential at −60 mV.

### 2.9 Simulations of 1C model

The membrane potential was simulated using a conductance based, leaky integrate and fire model in Matlab (version 7.3, Mathworks). The membrane equation was numerically integrated using the 4th-order Runge–Kutta method (Koch, 1999). No spiking mechanism was used, since the sole purpose of the model was to investigate sub-threshold fluctuations. Simulations were performed for time periods of 1 s using time steps of 0.05 ms. Standard deviation and integrated power was estimated and averaged over 25 simulations.

### 2.10 Power spectral estimation

The integrated power spectrum was estimated using the multi-taper method by Thomson (Thomson, 1982; Percival and Walden, 1998). The error bars on the power spectral estimation were assessed using a jackknife method. For the experimental data, 200 ms traces were selected from the on-cycles and off-cycles by a custom made procedure in Matlab (version 7.3, Mathworks). For the model and simulations 25 traces of 1 s each tapered with the first 5 Slepian functions (Percival and Walden, 1998; Berg et al., 2006). The power spectrum values were integrated in the frequency range from 25 to 80 Hz. We chose this region because this is the gamma frequency, which is often associated with network processing and gating (see, e.g., Cardin et al., 2009) and since previous work on motoneurons shows that *V_m_*-fluctuations are subject to intense increase in this spectral range during motor behavior (Berg et al., 2007). Furthermore, this range evades potential intrinsic resonant activity, which is almost entirely present at lower frequencies below 15 Hz (Jacobson et al., 2005) and instrument noise at higher frequencies. Our spectral estimation procedure has been uploaded to the Mathworks file-sharing database (http://www.mathworks.com/matlabcentral/) under the name Power spectral estimation with error bars.

### 2.11 Experiments

Experiments were performed for the purpose of extracting real values of synaptic time constant, conductance, and *V_m_*-fluctuations during different networks activity, to use as parameters in the model simulation and for general comparison.

#### 2.11.1 Integrated preparation

Red-eared turtles (*Trachemys scripta elegans*) were immerged in crushed ice for 2 h to ensure hypothermic anesthesia. Animals were killed by decapitation and blood substituted by perfusion with a Ringer solution containing (mM): 120 NaCl; 5 KCl; 15 NaHCO_3_; 2 MgCl_2_; 3 CaCl_2_; and 20 glucose, saturated with 98% O_2_ and 2% CO_2_ to obtain pH 7.6. The carapace containing the D4–D10 spinal cord segments was isolated by transverse cuts and removed from the animals, similar to studies published elsewhere (Keifer and Stein, 1983; Alaburda and Hounsgaard, 2003). The surgical procedures complied with Danish legislation and were approved by the controlling body under the Ministry of Justice.

#### 2.11.2 Recordings

Intracellular recordings in current-clamp mode were performed with an Axoclamp-2A amplifier (Axon Instruments, Union City, CA). Glass pipettes (part no. 30-0066, Havard Apparatus, UK) were pulled with an electrode puller (model P-87, Sutter instrument co., USA) and filled with a mixture of 0.9 M potassium acetate and 0.1 M KCl. Intracellular recordings were obtained from neurons in segment D10. Recordings were accepted if neurons had a stable membrane potential more negative than −50 mV. Data were sampled at 20 kHz with a 12-bit analog-to-digital converter (Digidata 1200, Axon Instruments, Union City, CA), displayed by means of Axoscope and Clampex software (Axon Instruments, Union City, CA), and stored on a hard disk for later analysis. Hip-flexor nerve activity was recorded with a differential amplifier Iso-DAM8 (WPI) using a suction pipette. The bandwidth was 100 Hz–1 kHz.

#### 2.11.3 Activation of network

Mechanical stimulation was performed with the fire polished tip of a bent glass rod mounted to the membrane of a loudspeaker in the cutaneous region known to elicit pocket scratch (Robertson and Stein, 1988). The duration, frequency, and amplitude of the stimulus were controlled with a function generator. This tactile stimulus induced the scratch-like network activity, which was monitored by the suction electrode nerve recordings from the Hip-flexor nerve.

#### 2.11.4 Slice preparation

Experiments were performed *in vitro* on transverse slices (0.3–3 mm thick) from the spinal cord lumbar enlargement (D8–S2) from the adult turtle (*Chrysemys scripta elegans*). The turtles were anesthetized by intravenous injection of propofol (0.1 mg/100 g) and killed by decapitation. The surgical procedures complied with Danish legislation and were approved by the controlling body under The Ministry of Justice. Experiments were performed at room temperature (20–22°C) in same Ringer solution as in the integrated preparation. Whole cell patch-clamp recordings of ventral horn interneurons were performed with borosilicate pipettes filled with Mg-gluconate (1.53 mM), MgCl_2_ (3.7 mM), CgCl_2_ (300 nM), HEPES (5 mM), Na-HEPES (5 mM), Na2ATP (2 mM), K-CH_3_SO_4_ (127 mM), and biocytin (10 mM). The pipette resistance was typically 5–10 MΩ when measured in the bath. Voltage-clamp recordings were performed with a Multiclamp 700B amplifier (Molecular Devices, Sunnyvale, CA). Data were collected by means of pCLAMP software (Molecular Devices), sampled at 1020 kHz with a 16-bit A/D converter (Digidata 1200 or Digidata 1322A; Molecular Devices), and stored on a hard disk for later analysis. Membrane potential values were not corrected for liquid junction potential. Drugs. Fast synaptic inputs were eliminated by a mixture of 6-cyano-7-nitroquinoxaline-2,3-dione (CNQX; 25 μM; Tocris), d-2-amino-5-phosphonopentanoic acid (d-AP5, 50 μM; Tocris), gabazine (100 μM; Tocris), and strychnine (10 μM) added to the extracellular medium. Interneurons were used as a surrogate for MNs, since we were unable to obtain whole cell patch recording on MNs.

## 3 Results

The membrane potential in neurons fluctuates during network activity. In the present study we used measurements from a population of spinal motoneurons (MNs) in adult turtles for comparison with our computer model.

### 3.1 Experimental data

The parameters used in the model (input resistance, capacitance, and synaptic conductance) were based on a population of MNs (data not shown). The passive membrane conductance during quiescence was 64 ± 5 nS (mean ± standard error, *n* = 32 MNs). The capacitance was 806 ± 38 pF (mean ± standard error, *n* = 32 MNs). The synaptic parameters were measured with whole cell patch-clamp recordings of spinal interneurons. For excitatory synaptic input the synaptic time constant was 2.4 ms (median) and 3.8 ± 0.8 ms (mean ± standard error, *n* = 487 from one neuron). The peak conductance was 0.43 nS (median) and 0.50 ± 0.01 nS (mean ± standard error, *n* = 487 events, one neuron). For inhibition, the synaptic time constant was 5.5 ms (median) and 6.2 ± 0.2 ms (mean ± standard error, *n* = 180 measurements in one cell). The maximum conductance was 1.3 nS (median) and 1.3 ± 0.6 nS (mean ± SD, *n* = 180 measurements in one cell, data not shown).

**Figure 1:**
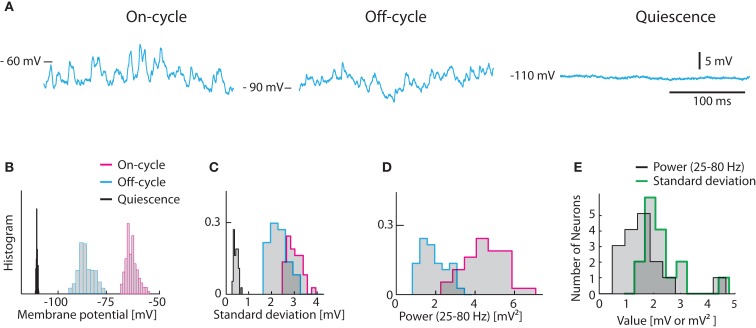
**Experimentally observed fluctuations in *V_m_* of turtle spinal motoneurons**. **(A)** Sample traces from a hip-flexor motoneuron during 3 states, on-cycle (left), off-cycle (middle), and quiescence (right) during application of a constant hyperpolarizing current of −2.5 nA to avoid spiking. **(B)** The distribution of *V_m_* for the traces in **(A)**. Histograms of standard deviations **(C)** and the spectral power in the 25–80 Hz band **(D)** for the same cells as in **(A,B)**. The spectral power of the quiescent state is not shown in **(D)** since it is insignificant. **(E)** Histogram of on-cycle average standard deviation and spectral power across the population of motoneurons.

The experimental data for conductance and *V_m_*-fluctuations during network activity was recorded from MNs during scratching (Alaburda and Hounsgaard, 2003; Stein, 2007). Scratching is a spinal network activity activated in the turtle by gentle touch within the appropriate receptive field on the carapace (Stein et al., 2005). The induced behavior consists of rhythmic contractions of the hindlimb muscles controlled by rhythmic bursting of their corresponding MNs. In our experimental preparation, the limbs and muscle were removed to secure stable intracellular recording of the MNs (Alaburda et al., 2005). Recordings from MNs during scratching revealed rhythmic synaptic input and phase related fluctuations of *V_m_* (see Figure 1).

The on-cycle refers to the phase of the rhythm where the MN would spike under normal conditions. We injected a negative constant current (of 2.5 nA) to prevent action potentials and measured the standard deviation as well as the spectral content in the gamma-band (25–80 Hz) for a 200 ms second window during the cyclic depolarizations. Similarly, we measured the membrane potential in the phase half-way between the rhythmic contractions, i.e., the off-cycle. In the off-cycle the membrane potential was more hyperpolarized and the fluctuations were always smaller (Figures 1A–C). For comparison, the membrane potential was recorded in the quiescence state before or several minutes after the scratch motor pattern. In this quiescent state *V_m_*-fluctuations were greatly reduced (Figures 1A–C). In addition, the spectral content in the gamma-band was orders of magnitude smaller than during on- and off-cycle (Figures 1A,D). The *V_m_* averaged standard deviation for the on-cycle for the population of MN ranged between 1 and 5 mV (mean = 2.13 mV, Figure 1E). The gamma-band spectral power was between 0.5 and 5 mV^2^ (mean = 1.44 mV^2^, Figure 1E).

### 3.2 〈*V_m_*〉 during concurrent inhibition and excitation

**Figure 2:**
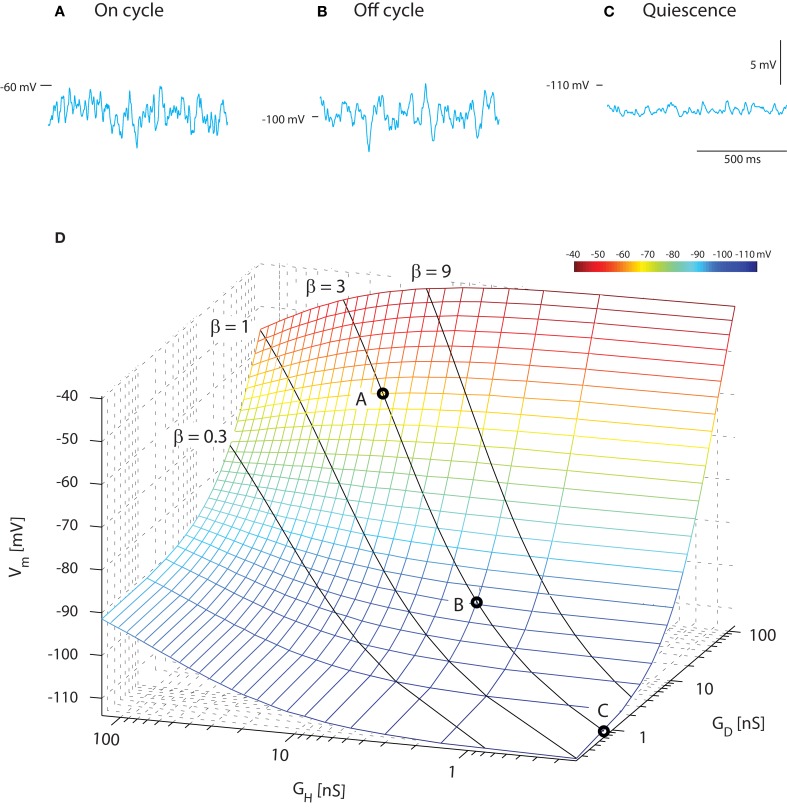
**Mean *V_m_* depolarizes during increase in concurrent inhibition and excitation**. Sample traces of membrane potential with synaptic input coming at a poisson stochastic rate (β = 1, i.e., *G_D_* = *G_H_* and *G_int_* = 0). The total synaptic conductances are 80 nS **(A)**, 12 nS **(B)**, and 1 nS **(C)** corresponding to the on-cycle, off-cycle, and quiescence states (Figure 1). **(D)**. Average membrane potential as a function of mean depolarizing and hyperpolarizing conductance. The solid lines show where the ratio of these is fixed (β = constant, indicated), but intensity varies along the line. The circled values along the β = 1 line denote the locations of the sample traces in above.

A constant ratio (β) between excitatory and inhibitory conductance does not imply that the mean membrane potential is constant. In fact, it would be possible to get rhythmic depolarizations in the membrane as observed in, e.g., motor behavior and locomotion not only as traditionally assumed by reciprocal inhibition and excitation, but also by rhythmic increase in concurrent inhibition and excitation (Berg et al., 2007). In order to illustrate this counter-intuitive fact and compare with the experimental result of Figure 1, we performed a heuristic testing of the behavior in the model with concurrent and Poisson-distributed inhibition and excitation. The average membrane potential for the on-cycle, off-cycle, and quiescence states could be recreated in the model (sample traces, Figure 2A) using the same current injection (−2.5 nA) that was applied in the experiments (Figure 1A). For simplicity we keep both *G_int,H_* = *G_int,D_* in order to focus on the excitatory (*G_D_* = *G_Syn,D_*) and the inhibitory conductance (*G_H_* = *G_Syn,H_*) relation with 〈*V_m_*〉. The on-cycle data could be recreated with high input intensity (*G_D_* = 60 nS and *G_H_* = 20 nS, β = 3) resulting in a membrane potential with mean −63 mV and standard deviation 1.3 mV (Figure 2A). The off-cycle data was recreated with a lower input intensity (*G_D_* = 9 nS and *G_H_* = 3 nS, β = 3) resulting in a 〈*V_m_*〉 = −100 mV and σ = 1.2 mV (Figure 2B). Finally, the quiescence state could be recreated with close to zero intensity (*G_D_* = 0.72 nS and *G_H_* = 0.24 nS, β = 3) resulting in a membrane potential with mean −113 mV and standard deviation 0.4 mV (Figure 2C).

As expected (see Section 4), varying the ratio between inhibition and excitation results in different levels of 〈*V_m_*〉 for different intensities. When β was kept constant and the input intensified, an increase in 〈*V_m_*〉 was observed. This is expected as the relative weight of the leak conductance declines, when the conductance from synaptic input increases (solid lines, Figure 2D). Values of 〈*V_m_*〉 in agreement with experimental data (cf. Figures 1 and 2) were obtained with an excitatory synaptic conductance three times larger than the inhibitory conductance (β = 3). This heuristic approach illustrates that for choices of parameters (β, inhibitory and excitatory conductances) with the constraint of constant ratio of inhibition and excitation (β) we are able to recreate mean and variance of *V_m_* that resembles those observed in experiments (cf. Figures 1 and 2).

### 3.3 Fluctuation in *V_m_* from synaptic conductance and no intrinsic conductance

**Figure 3:**
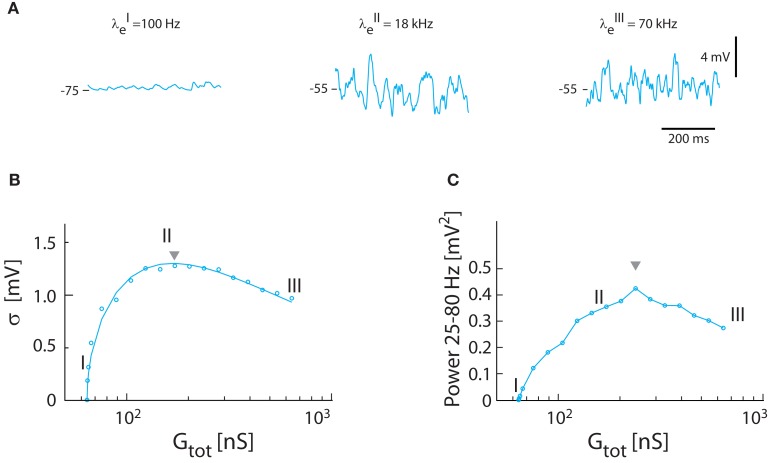
**Synaptic fluctuations in the model (γ = 1)**. **(A)** The membrane potential for three different intensities of excitatory and inhibitory synaptic input coming at a Poisson stochastic rate. The synaptic conductance is 0.3 nS (I), 108 nS (II), and 576 nS (III). The corresponding input rates are *λ_E_* = 0.1 kHz, *λ_I_* = 0 kHz (I), *λ_E_* = 18 kHz, *λ_I_* = 3 kHz (II), and *λ_E_* = 70 kHz, *λ_I_* = 20 kHz (III). Note that (I) is not balanced because the conductance is not large enough to depolarize *V_m_* to −55 mV, whereas (II) and (II) has reached balanced state, i.e., 〈*V_m_*〉 = −55 mV. **(B)** Standard deviation of *V_m_* as a function of total conductance, which is proportional to synaptic conductance. The roman numerals I, II, III denote the locations of the sample traces from **(A)**. Solid traces show theoretical values, and circles simulation results. **(C)** The spectral content in the gamma-band (25–80 Hz) as a function of total conductance.

Next, the relation between synaptic input intensity and fluctuations was tested in the model with balanced and Poisson-distributed inhibition and excitation, in the absence of intrinsic conductance, i.e., γ = 1. The magnitude of fluctuations in *V_m_* depended on the frequency of synaptic input (Figure 3A). The standard deviation of *V_m_* peaked at moderate input rates (21 kHz total synaptic input frequency with λ*_e_* = 18 kHz and λ*_i_* = 3 kHz). As observed previously (Kuhn et al., 2004) the relation between standard deviation and synaptic conductance had a reverse ∪-shaped curve (Figure 3B). With the parameters from turtle MNs the highest standard deviation possible with Poisson-distributed inputs was 1.3 mV (see triangle, Figure 3B), significantly lower than the 2–5 mV observed experimentally (Figure 1E). Nevertheless, there was good agreement between the expected magnitude of fluctuations as determined by numerical integration of equations (15) and (16) and the simulated *V_m_* (cf. the continuous line and points in Figure 3B). The integrated spectral power (25–80 Hz) had a similar dependence on input frequency (Figure 3C), though the peak was shifted toward larger conductance (i.e., triangle in Figure 3C is right-shifted). The peak value of the spectral power was 0.42 mV^2^, which was lower than the experimental value (cf. Figure 1).

### 3.4 Fluctuations for a blend of synaptic and intrinsic conductance

**Figure 4:**
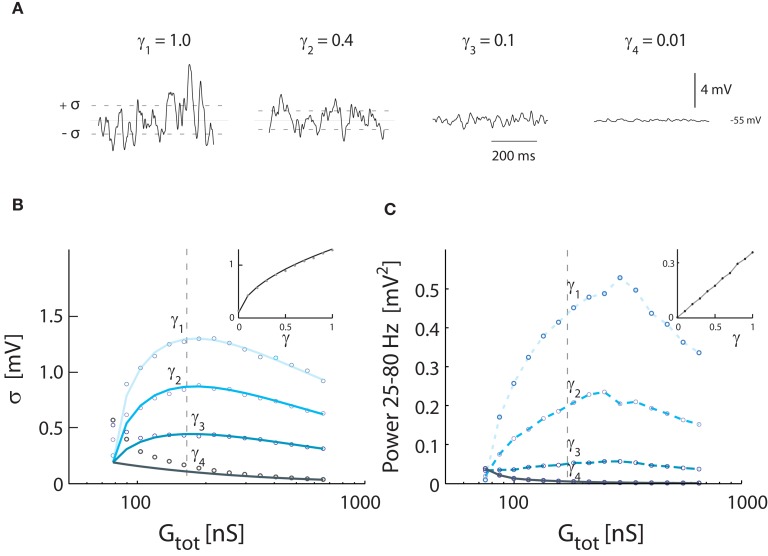
**Effects of inclusion of intrinsic conductance on *V_m_*-fluctuations, i.e., γ < 1**. **(A)** Sample traces of *V_m_* for different degrees of synaptic input, expressed in different values of γ (from top trace, γ = 1.0, 0.4, 0.1, 0.01). Total synaptic input rates (λ*_E_* + λ*_I_*) were 0.2 kHz (γ_4_), 2.5 kHz (γ_3_), 9.4 kHz (γ_2_), and 21 kHz (γ_1_). *G_tot_* was, for all traces, kept at the constant value (172 nS), which gave the largest variance in Figure 3. *V_m_* was kept balanced, i.e., 〈*V_m_*〉 = −55 mV. **(B)** The standard deviation of *V_m_* as a function of conductance, expressed as *G_tot_*, for different values of γ. The locations of the sample traces in **(A)** are indicated. Solid traces show theoretical values, and circles simulation results. Inset: The standard deviation at a fixed conductance (at the broken line, 172 nS) for different values of γ. **(C)** The spectral content in the gamma-band (25–80 Hz) as a function of conductance. The sample traces from **(A)** are indicated. Inset: Integrated power for at a fixed value of conductance (at the broken line, 172 nS) as a function of γ.

We tested how varying levels of intrinsic conductance affects *V_m_*-fluctuations at the same level of overall conductance. Fluctuations were largest for pure synaptic conductance (γ = 1). The fluctuations were progressively shunted as fluctuating conductance was replaced by non-fluctuating intrinsic conductance (Figure 4A). The standard deviation of *V_m_* and gamma followed the relationship (Figure 4B) described earlier [see equation (16)], dampened by $\sqrt{\gamma }$ [see equation (20) and inset Figure 4B]. Similarly, the spectral power as a function of increasing conductance had qualitatively the same shape as the standard deviation, but the peak was shifted to higher conductance (Figure 4C). The integrated power was linearly correlated with γ (see inset).

### 3.5 The impact of increasing synaptic coincidence

**Figure 5:**
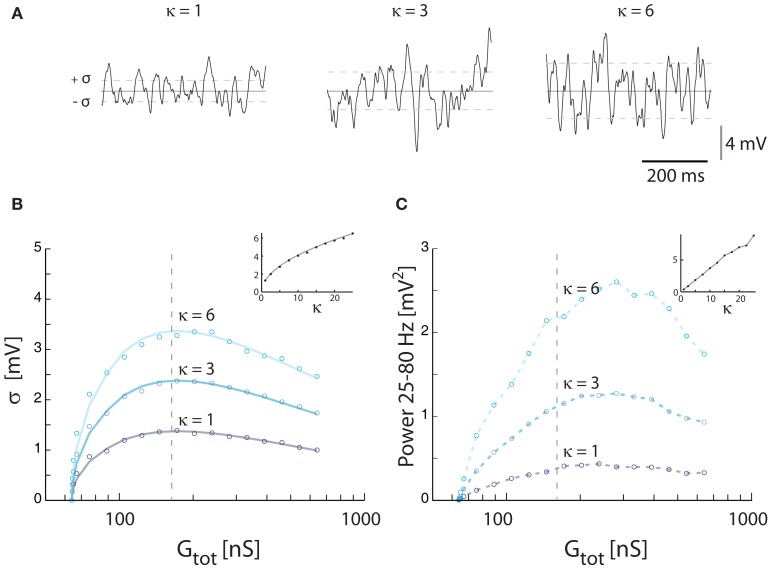
**Effect of correlated inputs with the synaptic conductance parameter fixed at γ = 1**. **(A)** Sample traces for membrane potentials for 3 levels of synaptic correlations. The total membrane conductance was kept at the conductance (172 nS) giving the largest fluctuations with synaptic input intensity at 21 kHz. *V_m_* was kept balanced, i.e., 〈*V_m_*〉 = −55 mV. **(B)** The standard deviation of *V_m_* as a function of synaptic conductance (expressed as total conductance) at different degrees of input coincidence (defined in κ). Solid traces show theoretical values, and circles simulation results. Inset: The standard deviation at a fixed conductance (172 nS) for different values of κ. **(C)** The spectral content in the gamma-band (25–80 Hz) as a function of synaptic conductance at different degrees of input correlation. The broken vertical lines in **(B,C)** are fiducials showing the locations of the traces in **(A)**. Inset: Integrated power for at a fixed value of conductance (172 nS) as a function of κ.

The magnitude of synaptic fluctuations, i.e., σ and the spectral power, had a reverse ∪-shaped curve as a function of input conductance and therefore as a function of synaptic input rate. However, the maxima of σ = 1.3 mV and power = 0.42 mV^2^ in the model (Figure 3) were substantially smaller than the values observed experimentally (Figure 1). As expected, the addition of intrinsic conductance (Figure 4) resulted in shunting, making it even more difficult to explain the large values of σ observed in experiments. For this reason we tested how coincident synaptic input affected fluctuations in the model (Figure 5A). We found that synchronized inputs resulted in both higher standard deviation (Figure 5B) and more spectral power (Figure 5C) than for purely uncorrelated Poisson input (cf. Figure 3). The relationship between input conductance and the fluctuations had qualitatively the same reversed ∪-shaped curve. With a coincidence factor of κ = 6 the standard deviation peaked at 3.2 mV, which is comparable to the experimental results. Using the approximated relation between κ and ρ, κ = 6 corresponds to a correlation at the peak of ρ = 0.003 (1770 presynaptic neurons) for excitation and to ρ = 0.017 (299 presynaptic neurons) for inhibition, all assuming an average firing rate of 10 Hz per presynaptic neuron. The standard deviation of *V_m_* in the computational model (open circles, Figure 5B) followed the shape expected from equation (16) both as a function of κ [equation (21)] and as a function of conductance (solid curves Figure 5B).

### 3.6 Inverse relation between γ and correlation

**Figure 6:**
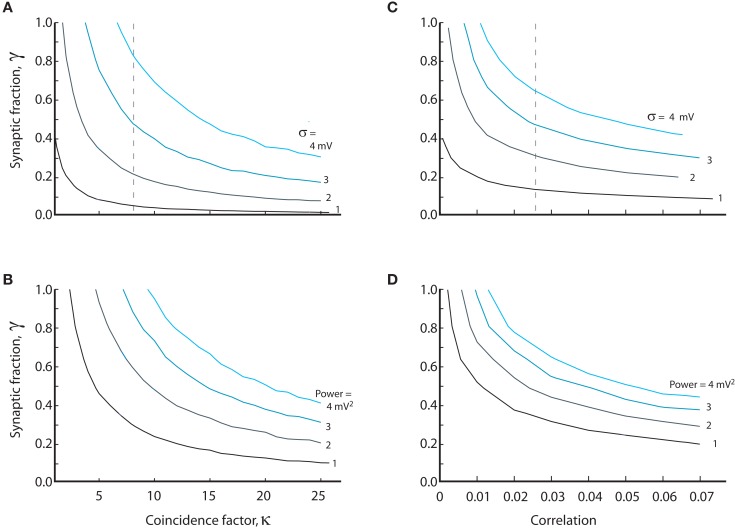
**The relation between synaptic coincidence factor (κ) and the synaptic to intrinsic conductance ratio (γ) for constant value of standard deviation (A) and amounts of power (B)**. Values on each curve are combinations of level of intrinsic conductance (γ) and coinciding input (κ) which give same standard deviation or power. **(C,D)** are the same as **(A,B)** except the abscissa is the correlation coefficient ρ (assuming average firing of 10 Hz in the presynaptic neurons) instead of the coincidence factor. The broken lines in **(A,C)** indicate the required coincidence/correlation for 50% synaptic conductance at 3 mV fluctuations. Equations (9), (11), (13), (18), (19), (23), and (24) were used to generate the data in this figure.

We investigated the opposing effects of intrinsic conductance and coincident synaptic inputs. At a fixed level of intrinsic and synaptic conductance (γ = *constant*), we estimated numerically the degree of coinciding input required to obtain a certain standard deviation and vice versa. The value of coincidence factor, κ, for a given value of standard deviation, was estimated as we changed the value of γ. The resulting relation was hyperbolic in shape with a cascade of curves for the increasing values of standard deviation (Figure 6A). The spectral power in the 25–80 Hz band was also integrated for different values of κ and γ. The curves for constant power (Figure 6B) had qualitatively similar shapes as the standard deviation. To compare to other studies we also show the curves based on an approximated input correlation measure (ρ; Figures 6C,D). Qualitatively, the curves follow the same hyperbolic shape as shown in Figures 6A,B. With moderate degrees of coincident input (κ < 7.5, ρ < 0.025) the conductance must be predominantly synaptic, i.e., γ > 0.5, to obtain standard deviations above 3 mV as observed experimentally (see broken line, Figure 6A). As for the standard deviation, the conductance must be predominantly synaptic (γ > 0.5) at moderate levels of coinciding input in order to obtain the power values of up to 4 mV^2^ observed experimentally (see broken line, Figure 6B). We note that the input must be correlated to obtain standard deviations above 2 mV, regardless of the degree of intrinsic conductance. This strongly suggests that synaptic input to MNs during network activity has to be correlated to obtain the values for the standard deviation observed experimentally.

### 3.7 BRK model

**Figure 7:**
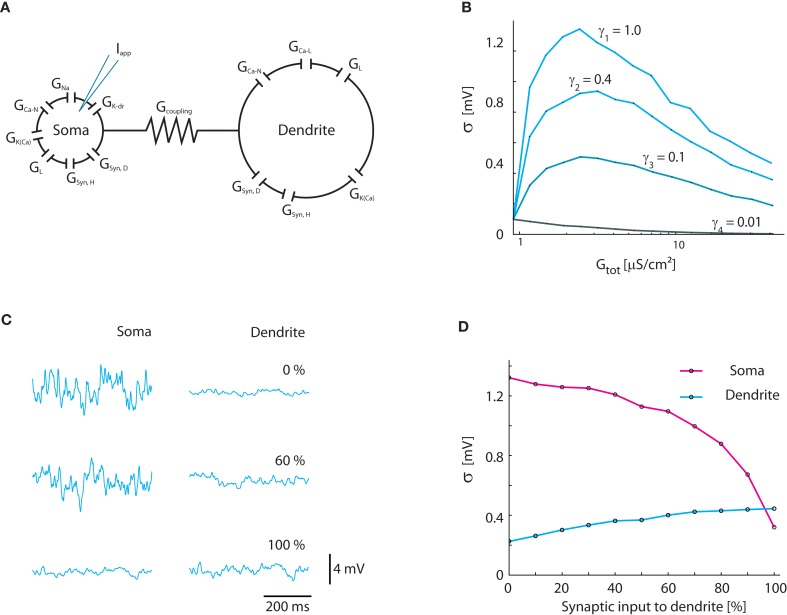
**Verification of results in two-compartment Booth–Rinzel–Kiehn model**. **(A)** Structure of Booth–Rinzel–Kiehn model with additional conductance-based synaptic input to soma and dendrite (*G_Syn,H_*, *G_Syn,D_*). **(B)** Effects of inclusion of intrinsic conductance on *V_m_*-fluctuations similar to Figure 3A. **(C)** Sample traces showing the effect of the distribution of synaptic input between soma and dendrite. *V_m_* was kept balanced, i.e., 〈*V_m_*〉 = −55 mV. **(D)** Standard deviation of soma and dendrite membrane potential at various distributions of synaptic input between the two compartments.

The results from the simple 1C model were confirmed with the established Booth–Rinzel–Kiehn two-compartment model of turtle MNs (Figure 7A). Similar to the result for the 1C model (Figure 4B), the maximum fluctuations occur at γ = 1 where the conductance increase is entirely synaptic (Figure 7B). At increasing conductance the magnitude of fluctuations reaches a maximum and starts to decline. We also tested the impact of how the synaptic input was distributed between the soma and dendrite compartment (Figures 7C,D). In agreement with the general functional properties of dendrites, the maximum fluctuations in the soma compartment are reached when all synaptic input is applied directly in the soma compartment (Johnston and Wu, 1995).

## 4 Discussion

During network activity the voltage dynamics of individual neurons is determined by their synaptic interactions and their intrinsic response properties. In MNs the relative weight of synaptic and intrinsic conductances during functional network activity is unknown. One can imagine two extremes: Neurons may have strong intrinsic dynamics making them largely autonomous entities coupled weakly through synaptic interactions (Grillner, 2006). Or the neurons receive massive synaptic input, which effectively overwhelms the intrinsic generated currents in controlling the membrane potential (Paré et al., 1998). Fortunately, these two mechanisms have contrasting effects on membrane potential fluctuations. While synaptic input induces *V_m_*-fluctuations, especially if correlated, slowly changing intrinsic conductances modulate mainly the mean value of *V_m_*. In the present study we use this discrepancy to estimate the relative contribution of intrinsic properties versus synaptic conductance. We model the *V_m_* using a conductance-based one-compartment model constrained by data from turtle MNs at rest and during network activity (Figure 1). We balance the potential at *V_m_* = −55 mV in order to minimize the number of parameters that would otherwise be necessary in our model to account for additional voltage dependent conductances. We verify the results in a two-compartment model with realistic intrinsic conductances (Figure 7). During scratching, MNs receive intense and concurrent inhibitory and excitatory synaptic input (Berg et al., 2007) which cause large *V_m_*-fluctuations and high input conductance (Berg et al., 2008). Both intrinsic and synaptic conductances contribute to high-conductance states (Stern et al., 1997; Steriade, 2001; Shu et al., 2003). However, statistical analyses of spike generation in turtle MNs during scratching did not detect a contribution of sub-threshold intrinsic response properties to spike patterns (Berg et al., 2008). In addition, the *V_m_*-fluctuations and high-conductance during scratching is voltage insensitive (Berg et al., 2007; Supplement). A reduced role of intrinsic properties in spike patterns during intense synaptic activity is well documented in other parts of the nervous system (Paré et al., 1998; Steriade, 2001; Fernandez and White, 2008, 2009; Riley et al., 2008). The fact that sub-threshold intrinsic properties have little or no role during network activity in the high-conductance state (Destexhe et al., 2003; Alaburda et al., 2005;Berg et al., 2007, 2008) suggests that spike generation is entirely dependent on the *V_m_*-fluctuations and the factors that influence the fluctuations. For this reason it is important to establish how *V_m_*-fluctuations depend on membrane properties, synaptic intensity, and level of correlation in the synaptic input. In our model the reverse ∪-shaped relation between the intensity of *V_m_*-fluctuations (Figure 3B) and synaptic intensity is shifted toward higher synaptic frequencies than originally observed by Kuhn et al. (Kuhn et al., 2004; Moreno-Bote and Parga, 2005). This is primarily due to higher resting conductance and lower unitary synaptic conductance in MNs than used in previous models. It is not known if variance and power of *V_m_*-fluctuations in motoneurons display inverted ∪-shaped curves with synaptic intensity during scratching. Qualitatively, however, average conductance, *V_m_*, variance and power of *V_m_* covary during scratching (Berg et al., 2007). This is compatible with a positive correlation between synaptic frequency and fluctuation, i.e., the left leg of the inverse ∪-shaped curves (Figure 3B). In functional terms it follows that spiking at membrane potentials near threshold scale with synaptic intensity (Arsiero et al., 2007). A similar positive correlation between synaptic frequency and *V_m_*-fluctuations was previously observed in neocortical neurons (Destexhe and Paré, 1999) and the shunting effect from synaptic input has been discussed elsewhere (Barret, 1975; Bernander et al., 1991; Borg-Graham et al., 1998; Chance et al., 2002; Berg et al., 2008).

Our results from modeling show that even in the absence of intrinsic conductance, uncorrelated synaptic activity cannot produce *V_m_*-fluctuations of the magnitude observed experimentally, i.e., σ ≈ 2–4 mV (cf. Figures 1 and 3). This leaves little room for intrinsic conductance to contribute significantly to the high-conductance state unless we allow large values for the synaptic correlation (κ > 7.5, ρ > 0.025, Figures 4–6). In a balanced network consisting of *N* neurons, the average pair-wise correlations scales as 1/*N* and are therefore generically small (Hertz, 2010). This taken together is indirect evidence for either relatively strong presynaptic correlation or a high intensity of synaptic input to MNs. In our models we have ignored burst firing as a source of correlated synaptic input. Although this is not entirely justified, burst firing in spinal interneurons during scratching have not been described (Berkowitz and Stein, 1994; Alaburda et al., 2005; Berkowitz, 2008).

The irregular firing of MNs during scratching (Berg et al., 2007, 2008) is in accord with previous assertions that highly variable spike timing in the high-conductance state is inconsistent with unbalanced random excitatory input (Softky and Koch, 1993; Shadlen and Newsome, 1998) and uncorrelated balanced synaptic input (Stevens and Zador, 1998; Harsch and Robinson, 2000; Salinas et al., 2000; Svirskis and Rinzel, 2000; Stroeve and Gielen, 2001). Thus our findings strongly support the view that irregular firing is indicative of a synaptic rather than an intrinsic generator of action potentials (Softky and Koch, 1993; Mainen et al., 1995; Shadlen and Newsome, 1998).

### 4.1 Synaptic strength

In the absence of experimental data and for simplicity we have chosen not to consider the effect of a broad distribution of synaptic strength on *V_m_*-fluctuations. Introducing a distribution of the synaptic strength will cause a distribution in the post-synaptic-potentials (PSPs). However, the effect is diminished for higher intensity input, due to summation of the PSPs. The variance of *V_m_* would be most affected at low intensity input, and converge toward the values of one mean synaptic strength for larger intensity input.

We were unable to obtain whole-call patch recordings from motoneurons in slices. For this reason we used the values for synaptic strength based on data from an interneuron in a slice experiment. The synaptic strengths in MNs could be larger than the estimates used in the present study. However, the impact of stronger synaptic connections on the model is similar to correlation among presynaptic neurons with lower synaptic strength. For instance, if the synaptic strength of a pre-motoneuron is doubled, this would be equivalent to having two pre-motoneurons firing in synchrony. Thus, since our results suggest that a κ > 7.5 is necessary in order to achieve the variance observed (Figures 4–6), our conclusion that the intrinsic conductance serves a minor role, remains valid even if the average synaptic strength was sevenfold larger.

A potential alternative explanation for the large synaptic fluctuations, observed in experiments, could be amplification of dendritic PSPs via voltage-activated intrinsic conductances. It is therefore important to consider computational effects of morphology of multi-compartments and their active propagation.

### 4.2 One- vs. multi-compartmental model

The one-compartment model is an incomplete representation of neuron morphology (see, e.g., Williams, 2004) but it captures many basic features including passive time constant and input conductance. The spatial distribution of synaptic activity in MNs and the related dynamics of cable structure during network activity is unknown. For this reason we have mainly used a 1C model to analyze the effects of synaptic conductance and synaptic correlations on the variance of *V_m_*. In the two-compartment BRK model the results were qualitatively similar, but the size of fluctuations was lower than in the 1C model. This is consistent with the passive properties of dendritic arborization largely acting as a low-pass filter that dampens the somatic fluctuations in membrane potential caused by distal synapses (Williams and Stuart, 2003). One-compartment neuron models have no electrotonic attenuation of synaptic potentials and therefore sets an upper bound on synaptic fluctuations when ignoring the effects of mutual shunting. Nevertheless, when the mutual shunting from a synaptic conductance is included, it makes a difference where the synaptic contacts are located and therefore there is a qualitative difference between one-compartment and multi-compartment models. Electrotonically close synaptic inputs have sub-linear summation whereas synaptic potentials on different dendritic arbors will summate more linearly at the soma due to the shielding resistance of the arbor (Spruston et al., 1999). Such an electrical shield between dendrites and soma combined with a strong active dendritic propagation, will also result in a current-based process rather than a conductance-based. Computationally, this would implicate an additive rather than a divisive interaction between synaptic and intrinsic currents, which would debilitate our model paradigm. Nonetheless, this situation is unlikely in our MNs because we found that the synaptic potentials are influenced by the imposed membrane potential (*data not shown*), i.e., the IPSPs are easily reversed and the EPSPs increase when injecting hyperpolarizing current, which reveal a conductance-based paradigm.

These opposing situations of either a near-linear and attenuated dendritic synaptic input or a sub-linear and un-attenuated synaptic input make it difficult to determine if our model is appropriate. Nevertheless, turtle MNs are relatively compact with most dendritic branches terminating at one length constant and the longest branches at two length constants (Svirskis et al., 2001). For this reason the one-compartment model with conductance-based synaptic input is likely to be a reasonable approximation. In this case, there is little room for intrinsic non-fluctuating conductance to contribute to the total conductance and *V_m_*-fluctuations of the magnitude observed experimentally must therefore rely on some degree of correlated synaptic input.

### Network architecture

In our experimental preparation, the motor network does not receive sensory feedback or extrinsic synaptic commands that could enforce synchrony. Synaptic correlation must therefore be an emergent property of the recurrent interactions between interneurons obtained by self-organizing principles within the network itself (Kuramoto, 1984; Takahashi et al., 2009). This organization could be both local and long-range. The spinal motor network for scratching has both local segmental connectivity and long-range inter-segmental connections. There are approximately 5 projecting interneurons for every MN (Nissen et al., 2008) and at most 25000 neurons in the scratch network (Walløe et al., 2011). If the excitatory long-range connections have feed-forward synapses on MNs and local inhibitory neurons then concurrent inhibition and excitation is a natural consequence during intense activity. A higher level of long-range drive will result in both more excitatory input to MNs as well as more local feed-forward inhibitory input. The intense synaptic input observed during motor network activity is characterized by concurrent inhibition and excitation such that the ratio of synaptic excitation and inhibitory conductance (κ) is approximately constant. A constant κ would give rhythmic depolarizations as synaptic intensity increases (Figure 2) in agreement with experiment (Figure 1). The functional benefit of this concurrent activity could be that firing and firing range is stabilized over a wide span of synaptic intensity (Chance et al., 2002; Berg and Hounsgaard, 2009). The balance between inhibitory and excitatory synapses observed morphologically in cat MN (Ornung et al., 1998; Kernell, 2006) may emerge from self-organizing principles within the network. Multi-unit recordings in this preparation may offer a unique opportunity to explore the spatial and temporal distribution of correlations among inhibitory and excitatory interneurons in a functional network and the mechanisms that govern correlations and concurrent inhibition and excitation.

### Spike generation during network activity

The high-conductance state during network activity compromises spike generation in motoneurons (Alaburda et al., 2005; Berg et al., 2008). At the same time the membrane time constant can decrease by an order of magnitude (Berg et al., 2008). Together this favors temporal coding and spike generation in response to fast depolarizing transients (Azouz and Gray, 2000). In agreement, we found that spikes in MNs during network activity are preceded by brief depolarizing transients (Berg et al., 2007, 2008). The results of the present study show that even for a constant depolarization and conductance the output spike pattern of motoneurons can be regulated over a wide range, purely by changing the precise timing of synaptic input and correlation patterns among pre-MNs. The short response time in a balanced network in the high-conductance state allows synaptic correlations to shift very rapidly (van Vreeswijk and Sompolinsky, 1996). Whether firing patterns are regulated by this mechanism during functional network activity can now be tested experimentally.

## Conflict of Interest Statement

The authors declare that the research was conducted in the absence of any commercial or financial relationships that could be construed as a potential conflict of interest.
